# Overt and Subclinical Hypothyroidism in the Elderly: When to Treat?

**DOI:** 10.3389/fendo.2019.00177

**Published:** 2019-03-22

**Authors:** Valeria Calsolaro, Filippo Niccolai, Giuseppe Pasqualetti, Alessia Maria Calabrese, Antonio Polini, Chukwuma Okoye, Silvia Magno, Nadia Caraccio, Fabio Monzani

**Affiliations:** ^1^Geriatrics Unit, Department of Clinical and Experimental Medicine, University of Pisa, Pisa, Italy; ^2^Neurology Imaging Unit, Imperial College, London, United Kingdom; ^3^Obesity Center at the Endocrinology Unit, Department of Clinical and Experimental Medicine, University of Pisa, Pisa, Italy

**Keywords:** hypothyroidism, elderly, treatment, L-thyroxin, frailty

## Abstract

Hypothyroidism is characterized by increased thyrotropin (TSH) levels and reduced free thyroid hormone fractions while, subclinical hypothyroidism (sHT) by elevated serum TSH in the face of normal thyroid hormones. The high frequency of hypothyroidism among the general population in Western Countries made levothyroxine (LT4) one of the 10 most prescribed drugs. However, circulating TSH has been demonstrated to increase with aging, regardless the existence of an actual thyroid disease. Thus, when confronting an increase in circulating TSH levels in the elderly, especially in the oldest old, it is important to carry an appropriate diagnostic path, comprehensive of clinical picture as well as laboratory and imaging techniques. In the current review, we summarize the recommendations for a correct diagnostic workup and therapeutic approach to older people with elevated TSH value, with special attention to the presence of frailty, comorbidities, and poly-therapy. The treatment of choice for hypothyroid patients is hormone replacement with LT_4_ but, it is important to consider multiple factors before commencing the therapy, from the age dependent TSH increase to the presence of an actual thyroid disease and comorbidities. When treatment is necessary, a tailored therapy should be chosen, considering poly-pharmacy and frailty. A careful follow-up and treatment re-assessment should be always considered to avoid the risk of over-treatment. It is important to stress the need of educating the patient for a correct administration of LT_4_, particularly when poly-therapy is in place, and the importance of a tailored therapeutic approach and follow-up, to avoid overtreatment.

## Modification of the Hypothalamus-Pituitary-Thyroid Axis in the Older People

In order to understand the modifications of thyroid axis, from hypothalamus to peripheral tissues, commonly observed during aging, it is noteworthy to briefly review the feed-back mechanisms that rules hormone secretion in young adults. Thyroid hormones are under the controls of TSH levels making the latter a sensitive marker of thyroid function. In this regard, circulating TSH levels in healthy subjects vary according to the circadian rhythm and respond with logarithmically variations to minor changes in serum FT_4_ and FT_3_ values (1). Thus, the occurrence of abnormal serum TSH in young adults may imply that serum FT_4_ and FT_3_ are not normal for that person (2). Accordingly, increased serum TSH values indicate a reduced thyroid function while lower TSH levels may underline a hyper-function of the thyroid gland (3). Apart from specific thyroid diseases that may involve older people, the aging process *per se* plays a peculiar role on thyroid axis, from hypothalamus to peripheral thyroid hormone metabolism and action (4–6). The aging process leads to reduced iodine absorption and organification with an altered thyroid response to TSH. Moreover, changing in the TSH bioactivity, in the thyrocyte sensitivity to TSH, in thyroid hormone metabolism as well as in the receptors and co-factors modulating the response to T_3_ input has been described (7). Overall, these processes result in reduced thyroid hormone production (8–10). Interestingly, individuals older than 80–85 years presented a nocturnal surge of TSH partially or completely lost with attenuated inhibitory effect of corticosteroids thus, indicating an age depended hypothalamus impairment (2, 11, 12).

A more complex relationship between TSH levels and the aging process has been described in several observation studies even while excluding patients with thyroid disease or autoimmunity. In fact, some experiences (generally case-control) showed a trend toward lower TSH circulating levels in individuals older than 75–80 years and centenarians (4, 13), while more recent cohort studies demonstrated an opposite TSH level behavior during age with a shift toward higher values in older people. In particular, in subjects above 80 years of age, the upper limit of the 95% interval of confidence is around 6.0 mIU/L, reaching 8.0 mIU/L in over-90 s (14–16). Some authors interpreted the reduced TSH levels in centenarians as a central reset of thyroid function in order to prevent an excessive catabolism favoring “physiological aging” (4, 17). It is noteworthy to differentiate this possible physiologic condition from that observed in acute patients and/or in starvation where TSH and T_3_ levels are reduced while reverse-T_3_ (rT_3_) is increased and a poor prognosis *quoad vitam* and *quoad valetudinem* has been described (6, 18). In general, we could hypothesize that the aging process acts for an individual as it does a hypothyroid status resulting in a reduction of the basal metabolism (19). However, to date, on the basis of previous experiences, it is impossible to state if the described reset of hypothalamus-pituitary-thyroid cross-talk in the elderly (due to either reduced TSH secretion or thyroid hormones production) is an effect of the reduced metabolic status or a protective cause preventing the extreme catabolism that characterizes the aging process (19). In addition, when analyzing the aging process on thyroid gland we should mention that the prevalence of specific thyroid diseases increases with age (20) and subclinical thyroid dysfunctions are more frequent than overt diseases (7, 21). Consistently, the prevalence of subclinical hypothyroidism and the presence of autoimmunity against thyroid cells increases with aging (20), thus underling a possible immune mechanism age related that explain this finding.

Some experiences showed that the modifications of pituitary-thyroid axis during aging may have an impact on longevity (7) even if we should report that the most important results on thyroid hormones and lifespan regulation, were obtained in the studies carried out in centenarians (and almost centenarians) (20). In this regard, Atzmon et al. reported that disease-free population of Ashkenazi Jews were characterized by extreme longevity. In details, they have observed higher serum TSH level in centenarians as compared to the control group (younger unrelated Ashkenazi Jews) and also to another control group from The National Health and Nutrition Examination Survey (NHANES). Furthermore, the authors documented an inverse correlation between FT_4_ and TSH levels in centenarians and Ashkenazi controls. Another experience in this setting showed a possible thyroid genetic background associated to extreme longevity (22). In particular, two single nucleotide polymorphisms (SNPs) in TSH receptor (TSHR) gene (rs10149689 and rs12050077) correlated with increased TSH level in the Ashkenazi Jewish centenarians and their offspring (22). In line with this, a North Europe study (Leiden Longevity Study) confirmed the role of thyroid genetic background on lifespan regulation. Indeed, the offspring of nonagenarian population presented a low thyroid activity (reduced FT_3_ values) and a better metabolic profile compared to their partners with less long-lived parents (8).

Consistently, Corsonello et al. (23) demonstrated an inverse relationship between age and free thyroid hormones independently from TSH levels in a population of Southern Italy (23). Moreover, the offspring of oldest old people presented lower free triiodothyronine (FT_3_), FT_4_ and TSH levels when compared with age-matched controls (23). Another interesting finding related to the aging process and thyroid function were reported by Gussekloo et al. (24) who firstly showed in a cohort study that oldest old individuals with abnormally high levels of thyrotropin may have a prolonged life span (24). Interestingly, in animal models low levels of T_4_ were associated with extended longevity (25–29). For example, a very severe hypothyroidism leading to reduced core body temperature, substantially contributed to remarkable longevity in rodents (25).

A recent report from the Rotterdam study, including over 9,000 healthy home-dwelling subjects, does not confirm the increasing trend of TSH during age, showing instead a progressive reduction of mean serum TSH with a concomitant rising of anti-thyroid peroxidase autoantibody (TPOAb) values with increasing age (30) while the same group provided intriguing results on the peripheral FT_4_ values and outcome in the same cohort (31). Those with higher FT_4_ values at baseline presented a worse prognosis in term of frailty index (31). Consistently, other experiences in the elderly showed the importance of thyroid hormones peripheral values in term of clinical outcomes (32) reinforcing the hypothesis that, apart from TSH level in very old population, the peripheral pattern of FT_4_ and FT_3_ may also play a central role in the lifespan regulation at least in older population at risk of frailty (32).

## Epidemiology and Clinical Effect of Overt and Subclinical Hypothyroidism

Over the last decades, the demographic growth in the Occidental Countries determined an increase of the population over 65 years of age. In Italy, which is second only to Japan in the elderly population, the over 80 s are the 6.7% of the overall population, while 22% is constituted by >65 (33) Together with aging, the incidence of chronic diseases increase; thyroid disturbances are frequent among the elderly. Hypothyroidism is defined by an increased level of thyroid stimulating hormone (TSH), with reduced circulating levels of free triiodothyronine (FT_3_) and free thyroxine (FT_4_) while, subclinical hypothyroidism (sHT) by increased TSH values in the face of normal circulating FT_3_ and FT_4_ levels (13, 20).

Among the general population in Europe, the prevalence of hypothyroidism varies between 0.2 and 5.3%, while in the USA between 0.3 and 3.7%, this variation probably being due to different iodine intake in diverse areas (34). Many factors may affect the response to excess iodine, among them route and duration of intake, iodine bioavailability and the individual physiopathological status including age, previous iodine intake and thyroid health. Indeed, excess iodine may more likely induce thyroid dysfunction (mainly hypothyroidism) in older subjects with underlying thyroid disease and insufficient iodine intake, than in those who live in iodine-sufficient areas without thyroid disease.

According to the National Health and Nutrition Survey (NHANES III), the global prevalence of hypothyroidism is 4.6%, respectively 0.3% for the overt and 4.3% for the subclinical type resulting the most frequent endocrine disease in the elderly, with a greater prevalence for the female gender (11). In UK, the prevalence of hypothyroidism is around 3.5–5% (35). The prevalence of sHT is variable, depending on the cohort considered (20) and going from 7.5%, as shown in the Wickham study (35) to around 21% in women and 16% in men as shown in the Colorado study (36). As demonstrated by the NANHES III study, TSH circulating levels and anti-thyroid autoantibodies increase with aging; in this study, 14% of the population 85 years old or above had TSH levels higher than 4.5 mUI/L, especially in the female gender (11). In a British population of 6,000 subjects older than 65 years, the prevalence of hypothyroidism was 2%, while the prevalence of sHT was around 2.9%, lower than what found in literature (37). In the same geographic area, the prevalence of sHT in subjects older than 60 years was around 11.6% in females and 2.9% in males (38). The huge difference in the data for the same area after 10 years was theorized to be due to an improved screening campaign and education, together with earlier treatment (39). That explanation may be reasonable, especially considering that the Medicine Utilization Center demonstrated that LT_4_ is in the 10 more prescribed drugs in Italy, consistently with the worldwide projections (40).

Considering that hypothyroidism is associated with increased mortality as well as increased incidence of cardiovascular events and cognitive and functional decline, replacement therapy with LT_4_ is recommended (41). A population-based retrospective study evaluating more than 2,000 hypothyroid subjects older than 65 years was recently published; the results showed that such condition was independently associated with higher risk of all-causes mortality. In older population, LT_4_ replacement therapy was associated instead with a lower risk of mortality. The mortality rate for CVD was similar between the groups receiving or not receiving LT_4_ (41). The association between hypothyroidism and all-causes mortality found in that study was in line with a previous longitudinal study, in which the same association in older subjects was found (42), but inconsistent with other epidemiological studies (43–45). Thus, further large prospective, randomized controlled trials (RCT) are necessary to better evaluate the effect of hypothyroidism and LT_4_ replacement on cardiovascular and all-causes mortality in the elderly.

Caution needs to be taken, however, in case of subclinical hypothyroidism, in the diagnostic and therapeutic management, particularly in the oldest old (20). Large set of data are available in literature, from meta-analysis and trials, about sHT (46, 47) which, together with the 2013 ETA (European Thyroid Association) guideline for the management of subclinical hypothyroidism, splits the population into two groups, depending on the values of circulating TSH levels, between 4 and 10 mIU/L or above 10 mIU/L (48). Bearing in mind that the thyroid function changes with aging and TSH values tend to increase, it is important to differentiate the age-related modification from the actual gland dysfunction. The most frequent pathogenic mechanism of sHT in the elderly is Hashimoto's thyroiditis (3, 49), although other secondary causes, such as insufficient replacement therapy following surgical or medical procedures (i.e., thyroidectomy or radioiodine treatment) need to be always considered. Hashimoto's thyroiditis, in 90% of cases, has a positive titer of anti-thyroid antibodies [anti-thyroglobulin and/or anti-thyroid peroxidase autoantibodies (TgAb and TPOAb, respectively)]; nonetheless, thyroid tissue damage is supposed to be caused by CD8+ T-lymphocytes, rather than the auto-antibodies themselves (50). Positive anti-thyroid autoantibody titers may represent a useful information not only about the presence of autoimmune thyroiditis, but also about the chance of progression to overt hypothyroidism, which has an yearly incidence of 4.3% in TPOAb positive patients, compared to 2.6% in the negative ones (50, 51). When demonstrated, the monitoring of the titer of anti-thyroid antibodies doesn't add much information, since it varies with the TSH levels (52). In the NHANES III study, the cohort of 13,000 healthy subjects was regularly followed up; the repeated dosage of FT_3_, FT_4_, TSH, TgAb, and TPOAb, showed that 10% of the subjects were positive for TgAb and 11% for TPOAb (11). In the around 20% of cases of antibody-negative sHT individuals, the diagnosis would be supported by the presence of tissue inhomogeneity and hypo-echogenicity at the thyroid US scan (53). Another possible cause of hypothyroidism in the elderly is iatrogenic. Drugs interfering with L-thyroxin absorption, as well as drugs potentially damaging the gland tissue such as ß-blockers, interferon-α, interleukin-2, lithium, ethionamide, tyrosin-kinase inhibitors, and thyrostatic medications (methimazole, perchlorate, and propylthiouracil), could determine hypothyroidism. The drug-induced damage is usually transient, and a periodical monitoring of the gland function, at least twice a year, is recommended (49). It has been widely accepted that thyroid hormones play a role in the cardiovascular system, modulating the adrenergic system activity, regulating the vascular peripheral resistance and in the protein synthesis (7). Unfortunately, while the impact of sHT on the cardiovascular (CV) system among the young adult has been recognized, among the elderly is still a matter of debate (20, 41, 54) especially since no RCTs have been conducted so far evaluating the impact of LT4 therapy on CV outcomes. A recent study involving over 2,100 subjects longitudinally, aimed at identifying a possible relationship between sHT and metabolic syndrome in the elderly. In the population examined, TSH level above 10 mUI/L was associated with higher odds of prevalent metabolic syndrome (21); circulating TSH levels above 10 mUI/L have been demonstrated to increase the risk of heart failure (HF) as well (7). The Prospective Study of Pravastatin in the Elderly at Risk (PROSPER) showed an association between HF and sHT over a follow up period of 3.2 years, in a population of 70–82 years old subjects, for TSH circulating levels above 10 mUI/L, while no association was found below that threshold (54). A large meta-analysis confirmed the association between HF and TSH levels above 10 mUI/L (or below 0.10 mUI/L) (46). More conflicting results has been reported for the relationship between sHT and coronary heart disease (CHD), more consistent in the younger population (7) (55), although a large meta-analysis showed an increased risk of CHD events and mortality for TSH levels above 10 mUI/L, across 35 years follow up, also adjusting for sex and age (45).

Thyroid hormones play a role in few metabolic functions, such as thermo regulation, oxygen consumption, glucose uptake, contra-insular activity, cholesterol mobilization and low-density lipoprotein (LDL) receptors expression in the liver (56). It is common, in hypothyroidism, to find increased levels of cholesterol and its sub-fractions (57); part of that is related to reduced cholesterol clearance, due to a reduced expression of the LDL receptor gene. Increased level of triglycerides is also a common finding in overt hypothyroidism, generally unmodified in sHT, following a reduced lipogenesis and lipase activities (58). The role of thyroid homeostasis in the cognitive development is widely known and accepted; not completely clear is the effect of thyroid failure, overt or mild, in the elderly and the impact it may have on cognitive impairment (59). An increased risk of Alzheimer's disease development has been seen in women at the lowest (< 1.0 mIU/L) and highest (>2.1 mIU/L) tertiles of serum TSH concentration in the Framingham study (60). Other studies showed interesting results; in the Health, Aging and Body Composition study, the risk of developing dementia was higher in subclinical hyperthyroidism, but not in sHT subjects (61), results consistent with a previous meta-analysis of Rieben et al. (62). Another recent meta-analysis showed a significant relationship between higher levels of circulating TSH and impaired cognitive performance in younger population (< 75 years of age) (63). On the other hand, a longitudinal study conducted on a cohort of cognitively normal subjects aged 60–90 years didn't find any relationship between TSH and thyroid hormones and hippocampal atrophy or risk of developing dementia (64). The inconsistent results available despite the important role played by thyroid function raise the need of long-term longitudinal studies, involving elder population, including the oldest old.

## Hypothyroidism in the Elderly: When to Treat?

Consistently to the principle that the therapy of choice for glandular deficiency is the replacement therapy, for overt hypothyroidism the first choice is LT_4_ replacement, also in older patients (65). The appropriate treatment of hypothyroidism, dealing to the resolution of the disease, leads to the release of symptoms, such as fatigue, constipation, increased sensitivity to cold, muscle weakness, and increased weight; improvement has been demonstrated in cognitive executive and cardiovascular functions (66). When considering the treatment of sHT in the elderly (especially in those older than 75–80 years), the approach has to be more cautious. It has been demonstrated in several studies that LT_4_ replacement therapy should be started when the TSH values are above 10 mUI/L, this being considered the value above which the risk of health disorders rises (7, 21, 46, 54). However, it is important to keep an approach on a case-by-case basis; this is particularly important in patients with potential other cardiovascular risk factors, which could hide the symptoms and signs related to sHT, already potentially less evident. Among the older population, it is also important to evaluate the potential frailty and comorbidities (66), appropriately tailoring the therapy. On that note, the evaluation of TSH levels and the trend over time is crucial; the international guidelines have set the cut-off level to 10 mUI/L, double checked and confirmed over 3 and 6 months before commencing the treatment (48, 49). Other than the TSH levels, the clinician should check the clinical presentation with signs and symptoms before deciding for any therapeutic approach (48), bearing in mind that many symptoms are unspecific (i.e., fatigue, constipation, sleeping pattern alteration, and fatigue), especially in the elderly with comorbidities (32). A well-structured approach, including a multidimensional geriatric assessment (67), comprehends a wide evaluation, which includes laboratory tests (FT_3_, FT_4_, TgAb, TPOAb) and US scan, to identify potential causes of thyroid failure (gland atrophy or autoimmune thyroiditis), responsible for permanently increased TSH levels. Whilst TSH levels tend to increase with aging, usually they don't exceed 7–8 mIU/L (14). In addition to the laboratory and imaging evaluation, the collection of a well accurate pharmacological history for drugs potentially affecting the thyroid function, such as amiodarone, lithium etc., is very important.

**Figure 1:**
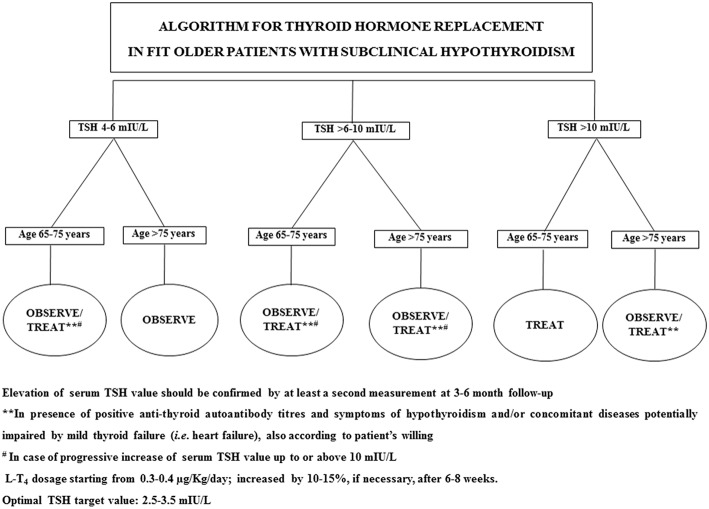
Suggested strategy of care according to either serum TSH value or the clinical features in fit older patients.

**Figure 2:**
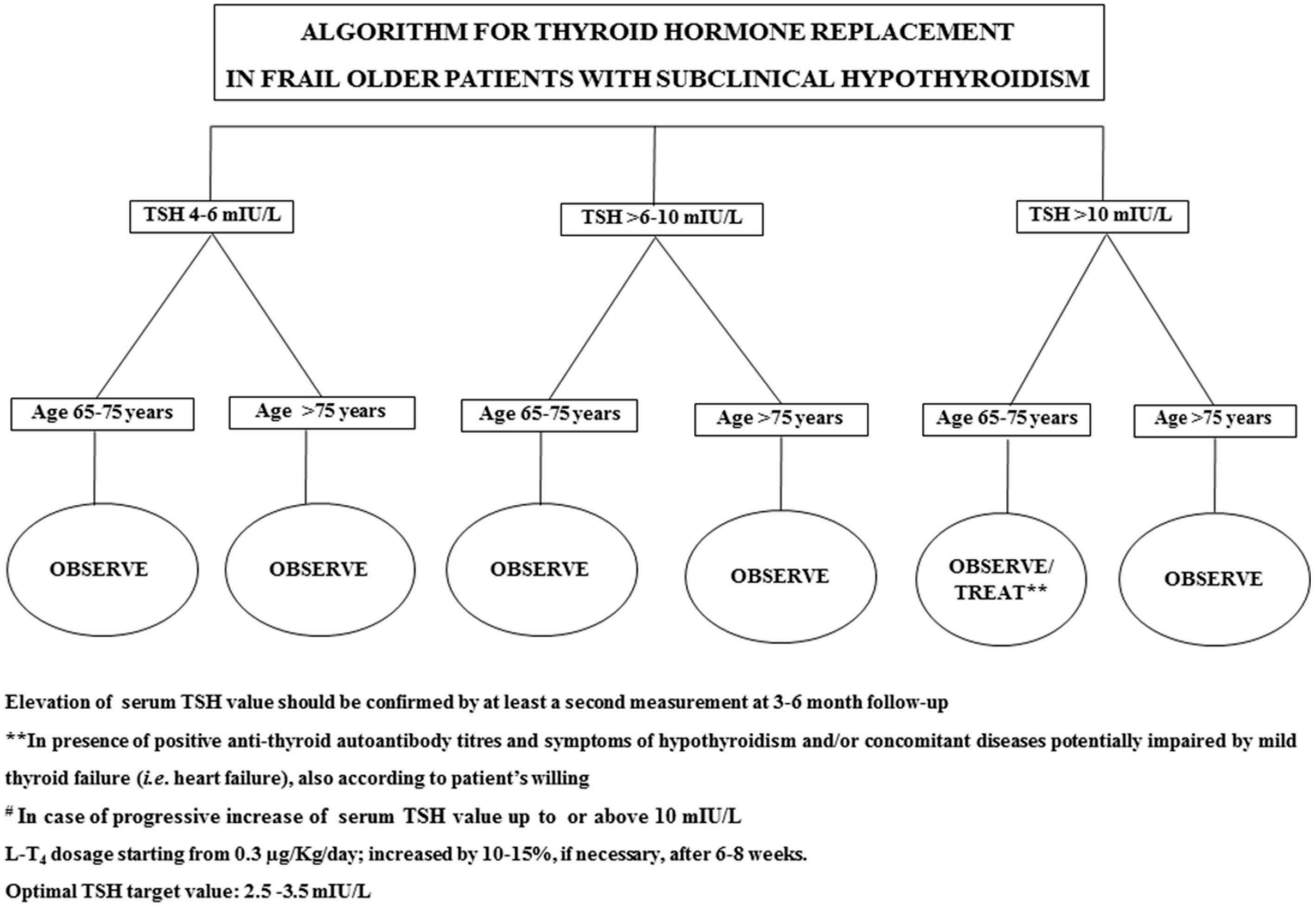
Suggested strategy of care according to either serum TSH value or the clinical features in frail older patients.

In 2017, Stott et al. conducted a double blinded, randomized, placebo-controlled study aiming to evaluate the efficacy of the therapy with LT_4_ on a large cohort (737 subjects) of older patient (mean age 74.4 years) with persistent sHT (mean entry TSH level: 6.40 ± 2.01 mIU/L) (68). The primary outcomes of the study were the changes, in 1 year, in the Hypothyroid Symptoms score and Tiredness score on a thyroid-related quality-of-life questionnaire. At the follow up evaluation, at 1 year, mean serum TSH level in the treatment group was 3.63 ± 2.11 and 5.48 ± 2.48 mIU/L in the placebo group. Among the groups, there were no differences in the quality of life measured with the questionnaire, nor difference in the adverse events of interest. The study concluded that the treatment with LT_4_ failed to provide an actual benefit in sHT subjects. However, some limitations in the study should be taken into account: serum TSH level at baseline was above 10 mUI/L only in few subjects, symptoms' level was low, and the presence of autoimmunity was not assessed. In particular, the latter limitation needs to be considered, since autoantibody positive patients are more likely to have progressive hypothyroidism, therefore long-term treatment could be actually beneficial (68). The study, moreover, was underpowered to detect the incidence of the LT_4_ therapy on cardiovascular events or mortality. Larger studies with a large cohort of older subjects with actual thyroid disease (i.e., with positive Ab titers) are not available at the moment. Our recommendation, in the elderly with sHT, is to approach the clinical management not only considering serum TSH levels and the 10 mUI/L cut-off, but also evaluating the presence of autoimmune thyroiditis as well comorbidities (especially HF) (69). On that note, the evaluation of the presence of frailty is crucial, considering how much impact it could have on the patient's quality of life and the clinical prognosis (32, 70): frail subjects are more likely to be affected by drugs side effect, and the risk of overtreatment, or poor compliance needs to be accounted in the clinical workup. The suggested clinical management of sHT in either fit or frail older patients is summarized in Figures 1, 2. In case of fit older (65–75 years) patients, LT_4_ replacement should be commenced when TSH levels are above 10 mUI/L (48, 49) while, fit oldest old (>75–80 years) should be treated when clear signs and symptoms of thyroid disease are present, after careful evaluation of cardiovascular and cognitive comorbidities; in absence of that, the strategy of choice should be the observation over time, in agreement with the ETA 2013 guidelines (48). A more cautious approach is suggested in frail elderly subjects, as shown in Figure 2. In frail subjects with TSH levels above 10 mUI/L, the wait-and-see strategy should be the one of choice, treating subjects in the 65–75 years of age range in presence of actual thyroid disease, symptoms of hypothyroidism and/or comorbidities potentially worsened by mild thyroid failure (i.e., heart failure). In case of serum TSH levels between 6 and 10 mUI/L, LT_4_ replacement therapy should be considered in “fit” subjects with risks factors for thyroid disease progression, such as anti-thyroid Ab, US pattern suggestive of disease, female gender; in absence of thyroid disease progression risk factors, an observation period with follow up of thyroid function every 3–6 months is suggested, commencing the therapy if the TSH level increases above 10 mIU/L (Figure 1). In the same range of values, but in frail subjects (Figure 2), the observation strategy is the one of choice. In frail patients younger than 75 years, with TSH levels below 10 mIU/L, the strategy of choice is to avoid LT_4_ replacement, unless the TSH level would progressively increase above 10 mIU/L during follow up, in presence of comorbidities potentially negatively influenced by mild thyroid failure, or in case of positive anti-thyroid auto antibody titers. In case of “fit” elderly younger than 75 years, with positive anti-thyroid autoantibody titer, symptoms of hypothyroidism and/or comorbidities influenced by mild thyroid failure, a trial with LT4 replacement should be considered (Figure 1). For all the subjects receiving replacement therapy, the titration of LT_4_ should be done from around 0.3–0.4 μg/Kg/day with increments by 10–15% after 6–8 weeks, if necessary, considering the optimal target values between 2.5 and 3.5 mIU/L, in agreement with international guidelines (48, 49). The regular monitoring and follow up of thyroid function is recommended over time, especially in the oldest old, to avoid over-treatment, which is known to negatively impact on cardiovascular an osteo-muscolar systems (48, 49).

Different formulations are available for the replacement therapy; the most used is the LT_4_ tablet, which is usually the first choice in absence of swallowing problems. However, considering the delicate process of absorption of the LT_4_, which can be influenced by several gastro-intestinal factors (71), some “rules” in regards of food and concomitant drugs administration should be followed (48, 49).

A debate is still open regarding the use of combined therapy T_4_+T_3_. Few studies have been conducted; most of the studies evaluated in a recent review of the literature failed to demonstrate a clear advantage with the combined therapy (72). The same lack of advantage over the monotherapy has been seen also in two different meta-analyses (73, 74). A large study evaluating the outcomes over 17 years follow up in population undertaking T_3_, mostly associated with T_4_, compared to a population receiving only LT_4_, didn't show any difference in the cardiovascular events, atrial fibrillation, fractures, diabetes mellitus or death; the group receiving T_3_ showed an increased rate of use of antipsychotic drugs (75). According to the 2012 ETA guidelines (10), the combined therapy T_4_/T_3_ should be used only in case of persistent complaint from the patient despite normal values of TSH with the monotherapy, after adequate education regarding the chronicity of the thyroid condition. Moreover, the combined therapy should be interrupted if the clinical improvement is not reached within 3 months (76). Thus, taking in mind the potential drawbacks of T_3_ therapy, the combined treatment with T_4_/T_3_ is generally not advised in older hypothyroid patients, especially in those older than 75 years.

Considering how variable the thyroid hormones could be among the general population, due to the influence of genetic, demographic (i.e., age and gender) and environmental factors, it is important to tailor and personalize the individual's treatment and follow up approach.

## Conclusions

Hypothyroidism, overt or subclinical, is a very frequent chronic disease among the older population; however, TSH circulating levels have been demonstrated to increase with aging, regardless the existence of an actual thyroid disease. For this reason, when confronting an increase in TSH circulating level in a patient older than 65 years of age, and even more carefully in the oldest old, it is important to carry an appropriate diagnostic path, comprehensive of clinical picture, laboratory tests, in particular checking for anti-thyroid autoantibodies, and US scan. Moreover, in the older population, the presence of frailty needs to be considered and addressed (77). The therapy of choice is hormone replacement with LT_4_, whichever pharmacologic form is more adequate, starting with a dosage of 0.3–0.4 μg/Kg/day and titrating by 10–15% after 6–8 weeks, aiming to keep an optimal TSH level of 2.5–3.5 mIU/L. It is important to stress the need of educating the patient for a correct administration of the therapy, particularly when poly-therapy is in place and the importance of a tailored therapeutic approach and follow up, to avoid overtreatment.

## Author Contributions

All authors listed have made a substantial, direct and intellectual contribution to the work, and approved it for publication.

### Conflict of Interest Statement

The authors declare that the research was conducted in the absence of any commercial or financial relationships that could be construed as a potential conflict of interest.
